# Flexible Sensorized Tube for Pipeline Defect Detection Based on Bending and Pressure Sensing

**DOI:** 10.3390/s26113400

**Published:** 2026-05-27

**Authors:** Yikang Chen, Hongyuan Chen, Yuan Yin, Junyi Chen, Bo Lu, Tao Chen, Minglu Zhu

**Affiliations:** 1 School of Future Science and Engineering, Soochow University, Suzhou 215123, China; 20234229006@stu.suda.edu.cn (Y.C.); 20234229037@stu.suda.edu.cn (Y.Y.); chent@suda.edu.cn (T.C.); 2 Jiangsu Provincial Key Laboratory of Advanced Robotics, School of Mechanical and Electric Engineering, Soochow University, Suzhou 215123, China; 20255229162@stu.suda.edu.cn (H.C.); 20245229045@stu.suda.edu.cn (J.C.); blu@suda.edu.cn (B.L.)

**Keywords:** flexible sensor, laser-induced graphene (LIG), pipeline defect detection, multimodal sensing, structural health monitoring

## Abstract

Urban pipelines are essential infrastructure components in modern cities. Their curved and confined structures make sensing difficult to achieve. In conventional flexible sensing devices, pressure and bending signals often interfere with each other. To address this problem, we propose an integration strategy for multi-array sensors on flexible printed circuits. The approach integrates laser-induced graphene pressure sensors and bending sensors on a polydimethylsiloxane substrate with flexible printed circuits. This integration enables stable and reliable signal acquisition and the device shows good performance under pressure loading. It has high linearity (R^2^ > 0.99), low hysteresis (2.68%), and a fast response time (~50 ms) in the range of 0–120 kPa. The sensing architecture is based on geometry-induced strain-field differentiation, which suppresses pressure–bending cross-interference and improves multimodal signal discrimination through structural design. Pressure mainly produces isotropic signals, while bending generates anisotropic strain signals. We test the device in simulated pipeline environments. Protrusion defects and corrosion defects generate different signal patterns. These differences allow clear defect identification. The device further supports spatial posture sensing and bending-state monitoring in complex curved pipeline conditions.

## 1. Introduction

Pipelines serve as critical lifelines for modern industrial infrastructure, enabling global transportation of water, oil, natural gas and other essential resources. Safe and stable pipeline operation underpins economic security, environmental protection and public safety [1,2,3,4,5]. Long-term service in harsh environments exposes buried or semi-exposed pipelines to thermo-mechanical stress, ground deformation and internal corrosion. Unmonitored degradation gradually causes structural distortion, wall thinning and eventual leakage or rupture. The severe ecological and economic losses from such failures underscore the urgent need for reliable pipeline inspection technologies [6,7,8,9,10,11].

To deal with these problems, structural health monitoring and non-destructive testing methods have been widely developed. Common methods include closed-circuit television, magnetic flux leakage, and ultrasonic testing, and they can detect basic defects [12,13,14,15,16,17,18]. For example, in-line inspection tools with inertial measurement units (IMUs) can improve trajectory tracking and bending strain monitoring [19]. Other methods, such as deep learning-based vision inspection and ultrasonic-guided waves, can further improve detection sensitivity and automation [20,21,22]. However, these systems usually measure only one parameter or need complex equipment, so they are not suitable for continuous sensing under confined, curved, and changing conditions.

Flexible sensing technologies have gained attention because they can fit curved surfaces and work in confined spaces [23]. These sensors convert mechanical signals, such as deformation, pressure, and vibration, into electrical signals through piezoresistive, piezoelectric, and triboelectric effects [24,25,26]. For example, piezoelectric sensors are good for dynamic signals and piezoresistive sensors are suitable for static and slow strain monitoring [27,28,29]. However, when different mechanical signals exist at the same time, such as pressure and bending, the signals can interfere with each other. This leads to signal coupling and reduces measurement accuracy [30]. To address signal coupling, various strategies have been explored from the perspectives of sensor design, system integration, and signal processing. Sensor design strategies include the development of gradient microstructured sensors that differentiate mechanical responses [31,32,33], such as pressure sensors with gradient spherical-cap structures that enhance sensitivity to weak dynamic signals under high static pressure backgrounds [34]. In addition, stimulus-selective materials, such as crystal acylhydrazone photoswitch systems, exhibit distinct responses to light and mechanical force, enabling differentiated mechanical deformation behaviors [35]. System integration strategies rely on multi-sensor arrays and robust encapsulation to enable parallel acquisition of multiple physical signals while maintaining stability under large deformation [36]. In addition, front-end signal-conditioning circuits based on oxide thin-film transistors provide high-gain and low-noise amplification, which improves the signal-to-noise ratio and suppresses common-mode interference [37].

Laser-induced graphene (LIG) is a laser-based fabrication technique that enables direct patterning of conductive graphene-like structures on polymer substrates without masks [38,39,40,41]. It provides a simple and scalable method for producing conductive patterns with high conductivity and good mechanical flexibility. Due to its direct writing process, LIG is widely used as a patterning approach for flexible electronic devices and sensing structures in complex environments.

**Figure 1:**
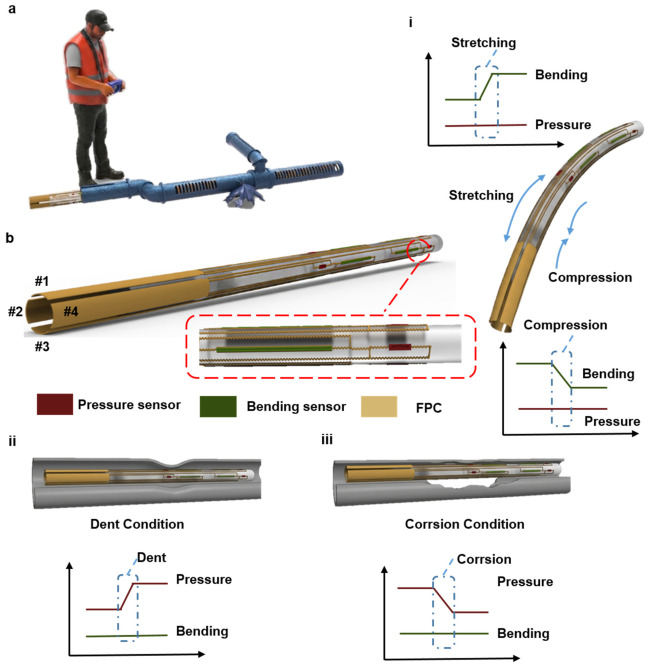
Design and sensing mechanism of the flexible pipeline monitoring device. (**a**) Schematic illustration of the device operating within urban pipelines for real-time structural inspection. (**b**) Structural configuration of the flexible sensing module, where an FPC integrating pressure and bending sensors is conformally attached to a tubular substrate and encapsulated with PDMS for mechanical protection and environmental stability. (**b**(**i**)) Signal response under bending deformation, showing distinct electrical variations induced by tensile strain on the convex side and compressive strain on the inner concave side. (**b**(**ii**)) Representative signal changes when the device passes through a dented pipeline section, indicating coupled responses to localized deformation. (**b**(**iii**)) Characteristic signal fluctuations during traversal of a corroded pipe region, reflecting irregular-surface-induced perturbations.

To enable real-time monitoring in complex urban pipelines, this work presents a compact flexible sensing device, as shown in Figure 1a. The device is designed for confined and variable geometries and allows in situ detection during movement. A flexible printed circuit (FPC) with pressure and bending sensors is attached to a soft tubular substrate and is encapsulated with polydimethylsiloxane (PDMS) so it can retain good mechanical strength and adapt to curved and irregular pipe surfaces, as shown in Figure 1b. The device enables dual-parameter sensing under typical deformation conditions. When bending occurs, the outer convex side is stretched and the inner concave side is compressed, so opposite electrical signals are produced and the bending direction and magnitude can be identified (Figure 1b(i)). When the device passes a dented region, local deformation causes coupled pressure and bending signals (Figure 1b(ii)). When it moves through a corroded region, irregular surfaces cause fluctuating signal changes (Figure 1b(iii)). By integrating pressure and bending sensors on a single FPC, the device can detect different pipeline conditions with a simple structure and reduce fabrication complexity while suppressing pressure–bending cross-interference and improving signal discrimination through multi-parameter correlation.

## 2. Materials and Methods

### 2.1. Design of the Soft Multimodal Sensing Tube

A silicone rubber tube (length: 230 mm, outer diameter: 10 mm, wall thickness: 1.5 mm) serves as the compliant backbone of the sensing device This elastomeric substrate balances structural rigidity and mechanical flexibility, supporting unobstructed navigation through confined, deformable pipeline spaces. Beyond mechanical support, the elastic silicone matrix efficiently transfers external deformations to embedded sensing elements, enabling stable electromechanical transduction.

Four FPCs are circumferentially distributed at 90° intervals around the scaffold, forming a 360° full-coverage sensing array. Each FPC (280 mm × 7 mm) integrates signal routing and dual sensing units, replacing discrete wiring with a compact, conformal network. Pressure and bending sensors within each FPC boost spatial resolution while minimizing radial profile, preserving navigability in narrow passages.

Both sensor types share a graphene–elastomer sandwich structure, with functional differentiation realized via geometric engineering of the conductive layer. Pressure sensors employ a circular graphene network embedded in PDMS; normal compressive loading rearranges conductive pathways, generating quantifiable resistance changes linked to local pressure. Bending sensors use elongated graphene microchannels (30 mm × 0.7 mm) with high aspect ratios, which concentrate directional strain under tubular bending. This geometry converts curvature into stable, high-resolution resistance shifts for spatial bending-state monitoring.

The combined design of the elastic scaffold, circumferential FPC array, and dual-mode sensing elements creates a mechanically coherent device. Strain-field differentiation driven by structural geometry facilitates discrimination between localized contact responses and global bending deformation signals. This structure-assisted signal differentiation strategy enables more reliable real-time monitoring in pipeline environments.

### 2.2. Sensing Mechanism of the Pressure and Bending Sensor

The sensor’s electromechanical response stems from dynamic modulation of a laser-induced graphene (LIG) percolation network embedded in an elastomeric matrix. The transduction relies on the microstructural rearrangement of graphene flakes, directly linking mechanical deformation to electrical signal variation. Sensor modality divergence arises from distinct network evolution patterns under different loading conditions.

Under normal compressive pressure, the porous LIG structure densifies as the PDMS layer deforms out-of-plane. New inter-flake contacts form and tunneling distances shorten, increasing percolation density and reducing resistance reversibly. This isotropic, contact-dominated behavior defines the pressure-sensing mechanism.

For bending detection, anisotropic strain redistributes across high-aspect-ratio LIG microchannels. Tubular curvature generates a strain gradient across the neutral axis, stretching and orienting graphene domains to disrupt conductive pathways selectively. This produces a monotonic resistance increase governed by strain-gradient effects.

Despite sharing the same LIG–PDMS composite, the two sensors primarily respond to distinct deformation mechanisms: isotropic pressure-driven densification versus anisotropic bending-induced strain redistribution. This geometry-assisted signal differentiation strategy reduces reliance on post-processing algorithms, enabling high-fidelity sensing in harsh operating conditions.

### 2.3. Preparation of Flexible Sensors

The pressure and bending sensors follow identical fabrication workflows, centered on LIG sensing layers encapsulated in elastic PDMS and bonded to conductive electrodes.

(1) LIG Formation on Polyimide Substrates

**Figure 2:**
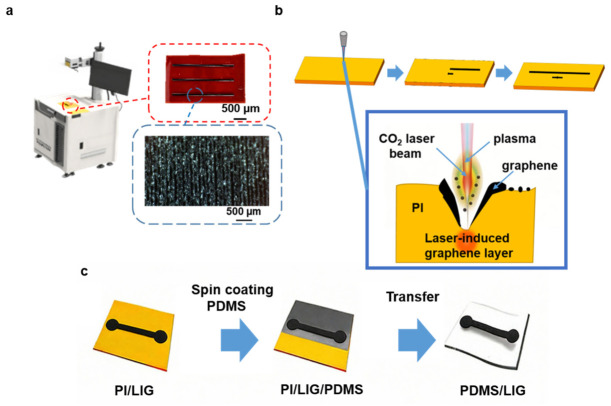
Fabrication and transfer process of LIG-based flexible sensors. (**a**) Laser-induced fabrication of LIG on a polyimide (PI) film. (**b**) Mechanism of photothermal conversion and graphitization during LIG formation. (**c**) Transfer process of LIG onto a flexible PDMS substrate.

Laser-induced graphene (LIG) is fabricated via a mask-free process that converts polymer precursors into conductive porous carbon networks. Polyimide (PI) films (250 μm thick; Suzhou Dongxuan Plastic Products Co., Ltd., Suzhou, China) serve as precursors due to strong mid-infrared absorption and efficient photothermal conversion under CO_2_ laser (λ = 10.6 μm; Suzhou Gaopusi Intelligent Equipment Co., Ltd., Suzhou, China) irradiation in Figure 2a. Laser exposure triggers photothermal decomposition, releasing heteroatoms and reorganizing residual carbon into a 3D interconnected LIG network with high surface area and strong electromechanical responsiveness, shown in Figure 2b.

(2) Optimization of Laser Processing Parameters for LIG Patterning

The laser parameters are optimized to ensure uniform, reproducible LIG formation over large sensing arrays. Use of a fixed focal distance (295 mm) maintains consistent energy density, with a laser power of 1.2 W, scanning speed of 300–400 mm/s, and line spacing of 0.02 mm. Pulsed irradiation (20 kHz, 20 ms delay) suppresses thermal accumulation, preventing PI ablation and preserving LIG network integrity.

(3) Transfer of LIG Patterns onto PDMS Substrates

To overcome PI rigidity, LIG patterns are transferred to compliant PDMS (Sylgard 184, Dow Corning, Midland, MI, USA), as shown in Figure 2c. PDMS prepolymer (base-to-curing-agent ratio 10:1) is spin-coated (500 rpm, 20 s) onto LIG-patterned PI and infiltrated under vacuum (−0.1 MPa, 30 min), forming robust mechanical interlocking. After thermal curing (90 °C, 1 h), the PDMS/LIG composite is peeled at 50–200 mm/min for damage-free transfer. A second PDMS layer is applied for full encapsulation.

(4) Integration of Multimodal Sensing Units into FPCs

Transferred LIG sensing elements are laminated onto FPC substrates (JLCPCB, Shenzhen, China) using silicone adhesive (E43, Wacker, Munich, Germany), preserving flexibility while ensuring mechanical stability. Lead wires are soldered to pre-patterned FPC pads, establishing low-resistance electrical connections for signal readout. Sensors are conformally assembled on the tube outer surface to maintain tight mechanical coupling. The full assembly is mounted onto inspection pipelines with UV-curable PI adhesive (ISITIC, Yantai, China), and a thin PDMS top layer provides final environmental and mechanical protection.

(5) Control System for Sensing

Pressure and bending sensors use voltage-divider circuits with 16 kΩ and 50 kΩ resistors, respectively. The signal-conditioning circuit was implemented on a custom printed circuit board fabricated by JLCPCB (Shenzhen, China). An AD8607AR (Analog Devices, Norwood, MA, USA) operational amplifier (op-amp) acts as a voltage follower for impedance matching, followed by a 10× gain stage to amplify weak signals. Conditioned signals are digitized by the analog-to-digital converter (ADC) integrated within the STM32 microcontroller unit (MCU; STMicroelectronics, Geneva, Switzerland), supporting real-time open-loop deformation acquisition.

### 2.4. Characterization Methods of the Sensing Units

**Figure 3:**
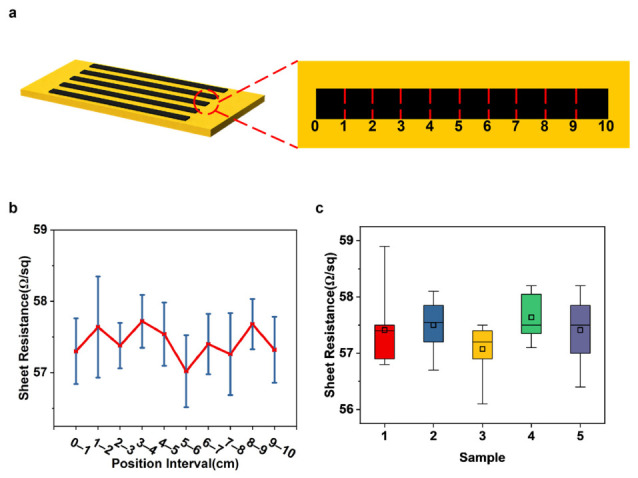
Fabrication reliability and macroscopic electrical uniformity of the LIG sensor arrays. (**a**) Schematic of the sheet resistance characterization setup. (**b**) Spatial mapping of sheet resistance across a 10 cm continuous array, divided into ten discrete intervals. (**c**) Boxplot analysis of five independent manufacturing cohorts.

Figure 3a illustrates the measurement of sheet resistance within a 10 × 1 mm^2^ rectangular region, which is uniformly divided into 1 × 1 mm^2^ square units. A total of five independent samples were prepared to ensure statistical reliability. The resistance of each unit was measured using a digital multimeter, and the corresponding sheet resistance was calculated to evaluate the spatial uniformity and consistency of the conductive layer.

**Figure 4:**
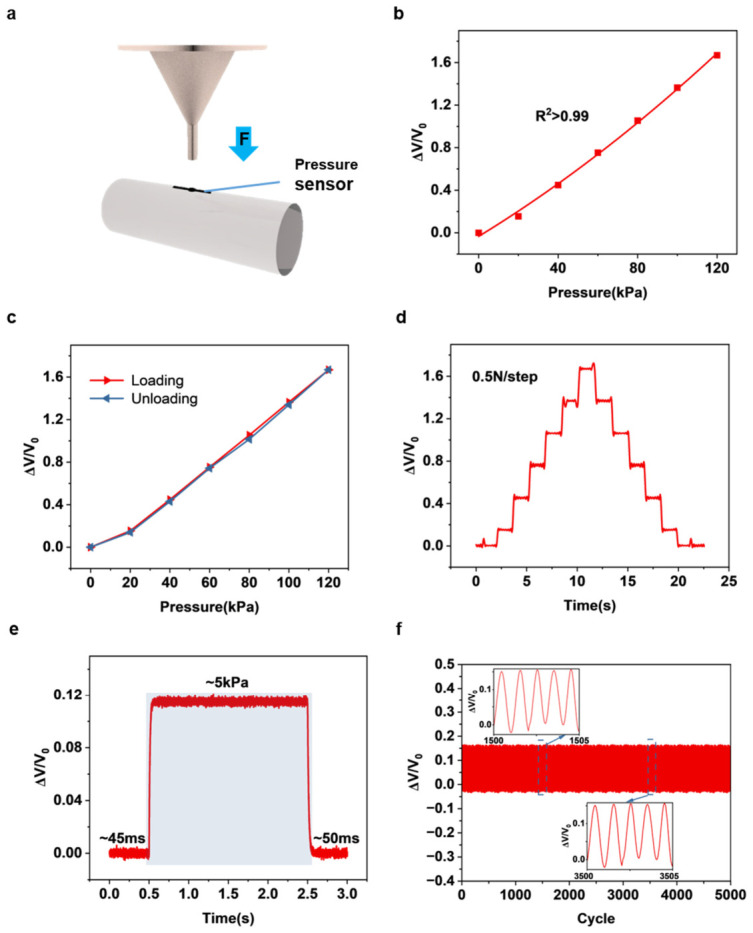
Characterization of the pressure sensor. (**a**) Experimental setup and schematic for evaluating the pressure-sensing performance on a pipeline-like structure. (**b**) Linear relationship between the relative signal change and applied pressure with high fitting accuracy. (**c**) Hysteresis characteristics of the pressure sensor during a loading–unloading cycle up to 120 kPa. (**d**) Real-time staircase response under incremental loading and unloading steps of 0.5 N per step. (**e**) Dynamic response and recovery characteristics under a transient loading pressure of 5 kPa. (**f**) Long-term durability demonstrated over 5000 loading–unloading cycles at 25 kPa.

Pressure sensor performance is evaluated using a MultiTest 2.5-i force-gauge system with a 3D-printed probe, as shown in Figure 4a. A commercial ILC 50N load cell records applied pressure, with probe area matched to the sensing region for uniform stress distribution.

**Figure 5:**
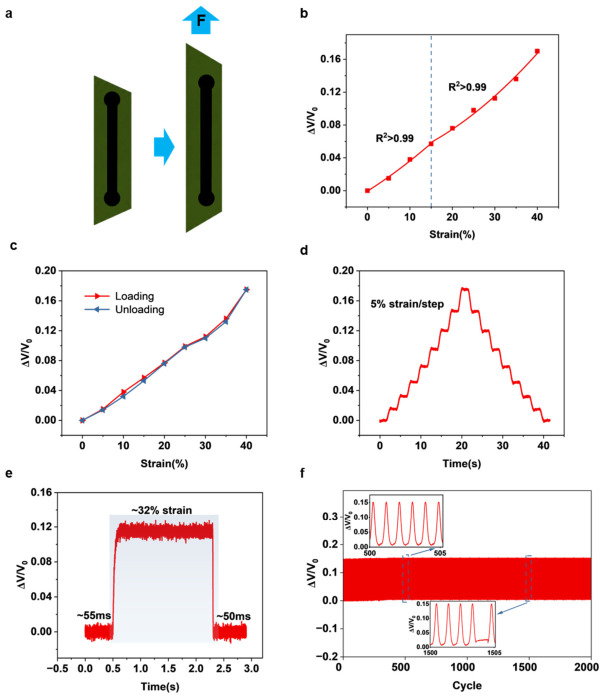
Tensile properties of the bending sensor. (**a**) Schematic of the characterization method; (**b**) relationship between the sensor’s output signal (ΔV/V_0_) and the tensile strain under loading conditions; (**c**) relationship between the bending sensor’s output signal and the tensile strain under loading and unloading conditions; (**d**) real-time monitoring of the output signal changes during one cycle of strain increase and decrease; (**e**) response and recovery times of the sensor; (**f**) repeatability test over 2000 cycles at a tensile strain of 38%.

**Figure 6:**
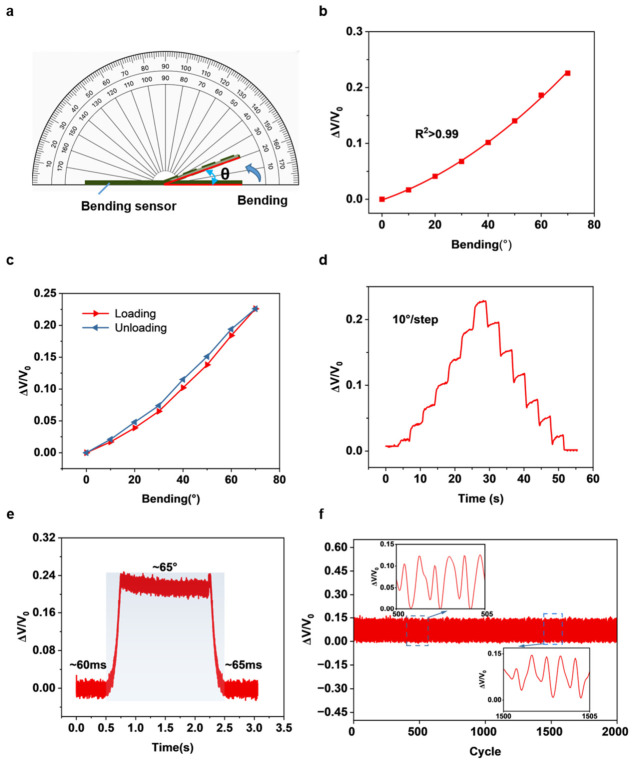
Characterization of the bending sensor. (**a**) Schematic of the experimental setup using a protractor for precise angle-dependent bending measurements. (**b**) Monotonic increase in the relative signal change as a function of bending angle from 0° to 70°. (**c**) Comparison between loading and unloading curves showing low hysteresis during a bending cycle. (**d**) Stepwise signal response of the bending sensor under incremental bending of 10° per step. (**e**) Transient response and recovery times of the sensor under sudden bending equivalent to ~65°. (**f**) Mechanical endurance and signal consistency over 2000 continuous bending cycles.

Bending sensors are characterized using a clamp-type tensile setup fixed to the force-gauge system, with tensile deformation applied at 50 mm/min, as illustrated in Figure 5a. As illustrated in Figure 6a, bending response is further quantified using a curved substrate with a back-mounted protractor, aligning the bending axis to the protractor center for accurate angle measurement. Electrical signals are recorded with a Tektronix MSO54B oscilloscope, with all tests repeated three times and averaged.

**Figure 7:**
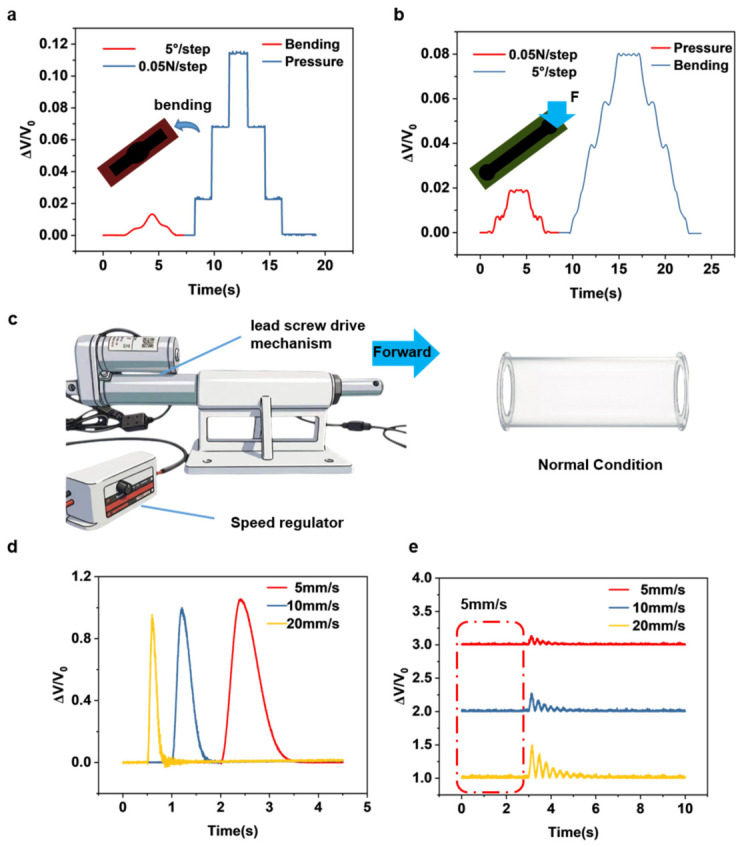
Signal differentiation and dynamic response analysis of the multimodal sensor. (**a**) Response characteristics of the bending sensing channel under bending and pressure loading. (**b**) Response characteristics of the pressure-sensing channel under pressure and bending loading. (**c**) Schematic illustration of sensor pulse behavior under different constant propulsion speeds during motion through a normal pipeline section. (**d**) Signal responses under different constant propulsion speeds. (**e**) Signal stability during speed transition tests.

**Figure 8:**
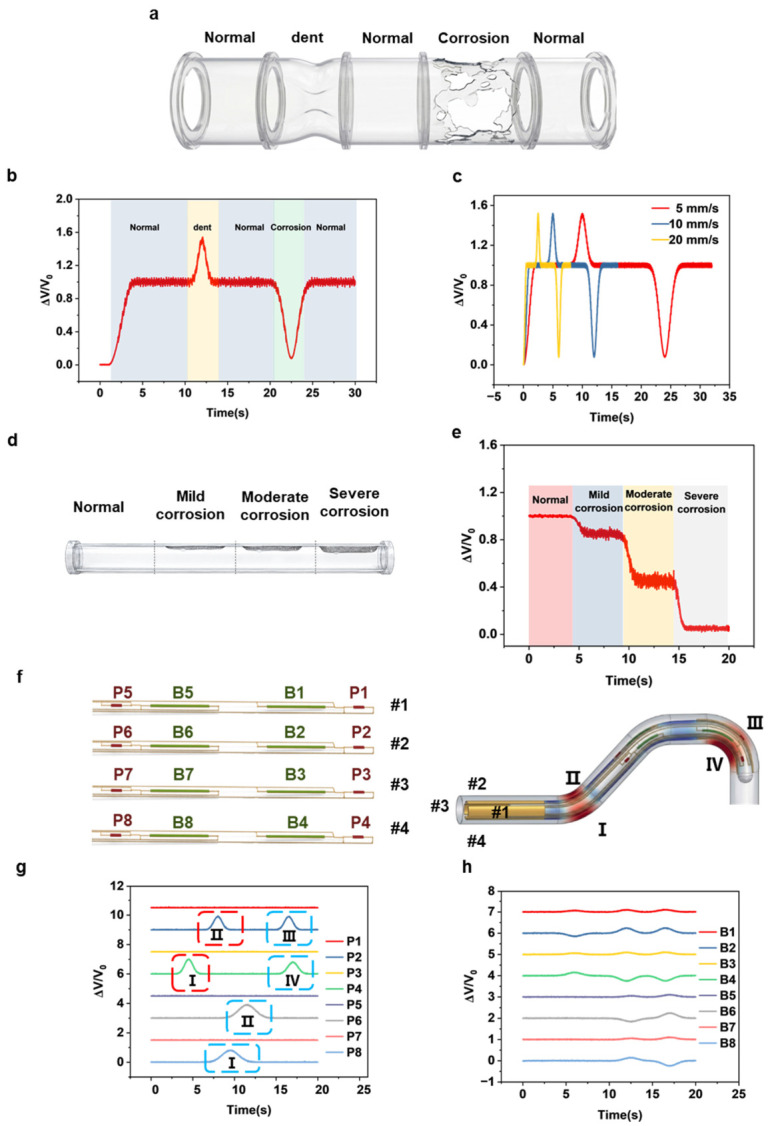
Comprehensive performance characterization of the flexible tube in complex pipeline environments. (**a**) Schematic of the pipeline model integrated with normal sections, dent conditions, and thinning corrosion features. (**b**) Real-time pressure signal responses. (**c**) Evaluation of signal robustness and sensitivity stability under varying propulsion speeds. (**d**) Schematic of the pipeline model with graded thinning corrosion features. (**e**) Real-time relative signal responses (ΔV/V_0_) corresponding to the graded corrosion conditions. (**f**) Spatial configuration and geometric layout of the multi-channel sensor array integrated on the flexible tube and S-shaped pipeline experimental model. (**g**) Spatiotemporal evolution of pressure signal streams during dynamic movement through the S-bend. (**h**) Spatiotemporal evolution of the bending signal stream during dynamic movement through the S-bend.

A system-level validation platform uses a speed-controllable electric linear actuator, 3D-printed connectors, and rigid fixtures to simulate pipeline locomotion. Device stability was tested in straight pipelines at varying translation speeds, as shown in Figure 7. The performance across different pipe surface conditions and speeds is shown in Figure 8a, while the bending-angle adaptability is evaluated in Figure 8d.

## 3. Results

### 3.1. Fabrication Reliability and Electrical Consistency

To evaluate fabrication reliability and large-area sensing consistency, the electrical uniformity and batch reproducibility of the LIG sensor arrays were systematically characterized. The spatial homogeneity of the LIG networks—critical for large-area distributed sensing—was assessed via sheet resistance measurements along a 10 cm continuous strip. Negligible standard deviation across the strip confirms the optimized laser graphitization process yields precise, long-range electrical uniformity essential for stable signal output.

Batch-to-batch reproducibility was validated across five independent fabrication runs in Figure 3b. As summarized by the boxplot in Figure 3c, the median sheet resistance values cluster tightly between 56.8 and 58.1 Ω/sq, with narrow interquartile ranges across all samples. These results reveal high structural consistency and process robustness, indicating LIG formation is insensitive to minor environmental fluctuations during photothermal conversion. The combined spatial uniformity and fabrication reproducibility indicate that the proposed laser processing strategy provides stable electrical performance and shows potential for scalable manufacturing of flexible sensing devices for intelligent pipeline inspection applications.

### 3.2. Characterization of the Pressure Sensing

The electromechanical performance of the LIG-based pressure sensors was characterized using a programmable force gauge with a custom probe. Over the 0–120 kPa range, the relative signal change ΔV/V_0_ increases monotonically with applied pressure, as shown in Figure 4b. The response exhibits high linearity (R^2^ > 0.99) with a sensitivity of 0.013 kPa^−1^.

The performance of the sensor is evaluated through a series of mechanical and electrical tests. The stability under repeated loading is examined first. During the loading and unloading cycles, the sensor shows low hysteresis of about 2.68%, as shown in Figure 4c. This behavior comes from the elastic property of PDMS and helps reduce signal drift caused by stress relaxation.

The sensitivity to small pressure changes is evaluated next. Step-response tests are carried out with load increments of 0.5 N. The signal forms clear and stable plateaus at each step, as shown in Figure 4d. This indicates that the sensor has good resolution for detecting small variations in pressure.

To evaluate the practical sensing resolution under realistic pipeline conditions, the pressure sensor was characterized under grease-coated conditions. As shown in Figure S2a, clear and stable signal plateaus were observed under incremental pressure steps of 50 Pa, indicating a practical pressure resolution of approximately 50 Pa. Baseline noise analysis under unloaded conditions further confirmed stable low-noise performance.

The dynamic behavior is then analyzed. Under a transient load of about 5 kPa, the sensor responds quickly. The response time is 50 ms and the recovery time is 45 ms, as shown in Figure 4e. These results show that the sensor can follow fast changes in contact during pipeline movement.

The frequency-dependent sensing performance was further evaluated under cyclic loading from 1 to 4 Hz. Representative response waveforms at different frequencies are shown in Figure S2c, wherein the sensor exhibits stable periodic outputs without obvious signal distortion. As summarized in Figure S2d, the normalized ΔV/V_0_ amplitude remains above 0.97 over the tested frequency range, with less than 3% variation, confirming robust dynamic sensing performance.

The long-term reliability was tested. The sensor was subjected to 5000 loading and unloading cycles at 25 kPa. The signal amplitude and baseline remained stable during the test, as shown in Figure 4f. The waveforms from the 1500th and 3500th cycles almost overlap, which shows stable performance over time and strong bonding between the LIG network and the polymer substrate.

To systematically evaluate the influence of environmental thermal fluctuations on sensor performance, the temperature drift characteristics of the pressure sensor were investigated. As illustrated in Figure S2b, the pressure sensor exhibits a linear negative correlation between relative voltage change (ΔV/V_0_) and ambient temperature over the range of 20–80 °C, which is attributed to the intrinsic semiconducting behavior of the LIG conductive network. A total relative voltage variation of approximately 5% was observed across the tested temperature range.

### 3.3. Characterization of the Bending Sensor

#### 3.3.1. Tensile Properties of the Bending Sensor

The tensile sensing performance was evaluated under controlled strain conditions using a programmable tensile tester. The relationship between relative voltage change (ΔV/V_0_) and applied strain is first examined. As shown in Figure 5b, the response presents two linear regions. When the strain is below 15%, the signal shows high linearity (R^2^ > 0.99). When the strain increases to 15–40%, the sensitivity becomes higher, and the gauge factor reaches 3.66, while the linearity remains high (R^2^ > 0.99).

The loading and unloading curves are further compared in Figure 5c. The two curves almost overlap, and the hysteresis is about 3.2%. The stability under repeated deformation was also evaluated. Cyclic tensile tests were carried out, and the signal remains stable during continuous loading, as shown in Figure 5d. The signal returns quickly to the baseline after strain release, which supports stable measurement.

The bending sensing channel was further characterized to evaluate its strain resolution and baseline noise performance under tensile deformation. As shown in Figure S3a, distinct and repeatable signal steps were obtained under incremental tensile loading, demonstrating reliable discrimination of small deformation variations. Stable response plateaus with low signal fluctuation were observed during each loading stage, indicating a practical strain resolution of approximately 0.5%. In addition, the enlarged baseline noise profile under unloaded conditions revealed only minor output fluctuations around the equilibrium state, confirming stable low-noise signal acquisition.

The dynamic response of the sensor was then tested. A step strain of about 32% was applied. The response time was about 55 ms, and the recovery time was about 50 ms, as shown in Figure 5e. These results show that the sensor can respond quickly to sudden deformation.

The long-term reliability was tested. The sensor was stretched for 2000 cycles at 38% strain. The signal amplitude and baseline remained stable during the test, as shown in Figure 5f. The waveforms from the 500th and 1500th cycles are almost identical.

To evaluate the environmental robustness of the proposed device, the temperature-dependent baseline drift was investigated. As shown in Figure S3b, when the ambient temperature increased from 25 °C to 75 °C, the bending sensor exhibited a relatively small resistance variation, with a total relative resistance change (ΔV/V_0_) of approximately 5%. This corresponds to a temperature coefficient of resistance (TCR) of approximately −0.1%/°C, indicating limited temperature-induced baseline drift within the tested range.

#### 3.3.2. Bending Properties of the Bending Sensor

The bending performance was tested over a range of 0° to 70°. This range matches common pipeline bending conditions. The relative signal change (ΔV/V_0_) increases as the bending angle increases, as shown in Figure 6b. The signal shows good linearity (R^2^ > 0.99). This is caused by uniform microcrack changes in the LIG network during bending. These changes create a stable relationship between the deformation and electrical signal.

The loading and unloading behavior were tested next. The two curves almost overlap, as shown in Figure 6c. The hysteresis is very small. This shows stable contact between the LIG and PDMS. Step tests were carried out with 10° increments. The signal forms fast and stable plateaus at each step, as shown in Figure 6d. There is no overshoot in the signal. This shows that the sensor can detect small changes in bending.

The dynamic response was tested after that. The sensor was subjected to fast bending. The response time was about 60 ms. The recovery time was about 65 ms, as shown in Figure 6e. These results show that the sensor can follow fast bending motion.

The durability was tested last. The sensor was bent for 2000 cycles. The signal amplitude and baseline remained stable during the test, as shown in Figure 6f. There was no clear signal change over time.

### 3.4. Signal Differentiation Performance and Dynamic Variable-Speed Characterization

The interaction between pressure and bending responses was evaluated under controlled mechanical inputs. The results are presented in Figure 7.

The response of the bending sensor is examined first. Step bending of 5° per step was applied. Step pressure loading of 0.05 N per step was also applied. The results are shown in Figure 7a. The sensor produces strong and clear signals during bending. The response to small pressure changes is much weaker. The resistance changes show different patterns under bending and pressure. These distinct response characteristics indicate reduced pressure interference in the bending sensing channel.

The response of the pressure sensor is then analyzed. Step pressure and bending inputs are applied, as shown in Figure 7b. The sensor shows stable and repeatable signals under normal pressure. The signal under bending follows a different pattern. This reduces the effect of overall pipe deformation and improves the identification of localized pressure variations.

The device’s behavior at different speeds is evaluated next. A lead-screw platform was used to control motion. The speed was changed from 5 mm/s to 20 mm/s. The results are shown in Figure 7d.

The signal pulse becomes narrower as the speed increases. The peak value does not change. The signal stability during speed transitions was also examined, as illustrated in Figure 7e. After switching from 5 mm/s to higher speeds, the baseline noise increases slightly due to mechanical vibration and friction changes. However, all channels quickly stabilize with manageable baseline shifts. These results validate the dynamic reliability of the LIG/PDMS sensing device.

### 3.5. Sensing Performance Validation in Complex Pipeline Environments

To assess the operational performance of the sensing device in complex pipeline environments, we built a comprehensive validation framework covering typical geometric defects and nonlinear paths. The corresponding experimental results and quantitative analyses are presented in Figure 8.

Structural feature recognition was first evaluated using the physical model in Figure 8a, which incorporates three representative conditions: straight segments, a dent condition, and wall-thinning corrosion to mimic real-world inspection scenarios. Based on the dynamic sensing profiles in Figure 8b, the pressure signal stabilizes at a baseline near 1.0 when the flexible tube travels through a healthy pipe section, maintained by radial pre-compression from the inner wall. Upon entering a dent region, reduced cross-sectional area elevates contact stress, pushing the signal to a characteristic peak around 1.5. In contrast, when the flexible tube passes through a corroded region with an enlarged diameter, there is no contact between the sensing elements and the pipe wall. The pressure signal quickly drops to near zero. These signal changes from 0 to 1.5 show that the sensors are very sensitive to radial deformation. They also provide clear numerical limits for detecting internal geometric defects.

To further establish the relationship between sensing output and defect severity, dented pipe sections with predefined dent depths of 0, 0.5, 1.0, and 1.5 mm were tested under the same insertion speed. As shown in Figure 8c, the peak normalized pressure response increased with increasing dent depth. This trend indicates that a deeper dent induces stronger radial compression on the flexible tube, resulting in a larger contact-response signal. Therefore, although the voltage signal does not directly represent the intrinsic stress or strain of the pipe material, the response amplitude can be correlated with dent severity through controlled calibration.

The sensing stability under different propulsion speeds was further evaluated in Figure 8c. As the speed increased from 5 mm/s to 20 mm/s, the signal pulse width decreased accordingly, while the peak amplitude remained nearly unchanged, indicating stable sensing performance under variable motion conditions.

To further investigate sensing behavior in curved pipelines, continuous bending experiments were performed using an S-shaped pipeline model, as shown in Figure 8f. Localized pressure increases were detected by specific pressure-sensing units when the probe contacted bending regions (Figure 8g). Simultaneously, the bending sensing array exhibited differential responses under tensile and compressive deformation states (Figure 8h). Similar delayed responses were observed in the rear sensing array as it traversed the same structure, demonstrating consistent spatiotemporal sensing behavior during navigation in complex curved pipelines.

### 3.6. Validation in a Simulated Pipeline Model with Representative Structural Defects

**Figure 9:**
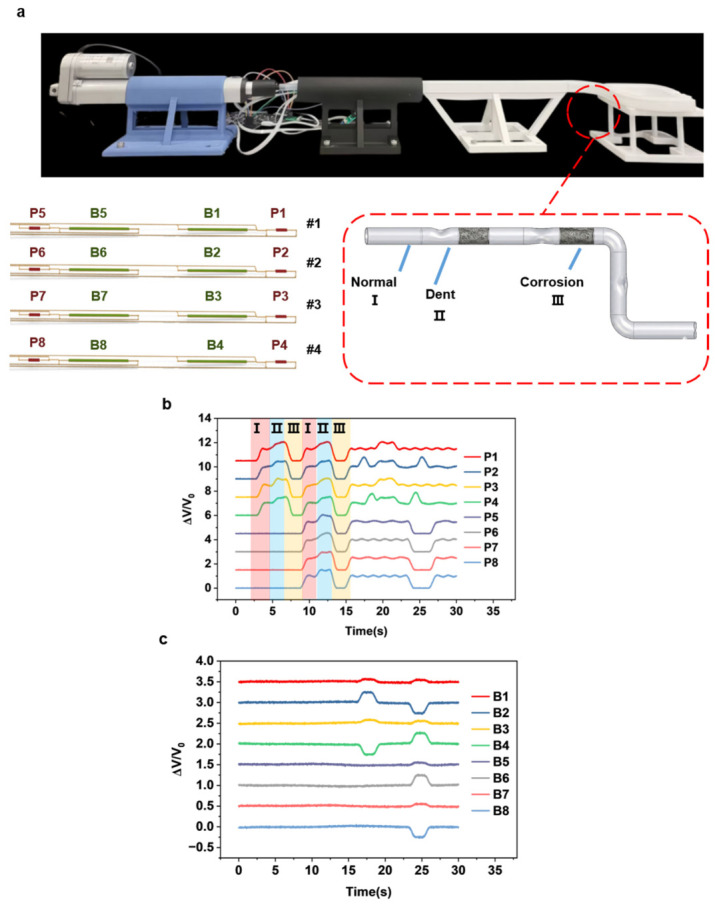
Experimental setup and real-time pressure and bending signal responses during dynamic pipeline inspection. (**a**) Schematic of the experimental platform. (**b**) Real-time response curves of the pressure sensor array. (**c**) Real-time response curves of the bending sensor array.

To evaluate the practical applicability of the flexible tube with integrated pressure and bending sensing units, a simulated pipeline inspection case was constructed, as shown in Figure 9. The pipeline model includes representative structural conditions, including normal pipe sections, dented/narrowed regions, wall-thinning corrosion regions, and locally curved/contact sections. During the test, the flexible tube was driven through the pipeline at a constant speed, while the pressure and bending responses were continuously recorded.

In the normal pipe section, the front pressure units (P1–P4) gradually increased from the initial unloaded state and stabilized at approximately 1.0 (ΔV/V_0_). This stable baseline response is attributed to steady radial contact between the flexible tube and the inner pipe wall. Meanwhile, the bending units (B1–B4) remained close to the baseline, indicating that the tube maintained an approximately straight posture with limited bending deformation. The signal responses changed distinctly when the tube passed through defect regions. In the dented/narrowed region, the local pipe diameter decreased, leading to enhanced radial compression between the tube and the pipe wall. As a result, the pressure signals increased rapidly and formed clear positive peaks of approximately 1.5 (ΔV/V_0_), as shown in Figure 9b. In contrast, the wall-thinning corrosion region produced an opposite response. Because local wall recession reduced or eliminated contact support between the sensing units and the pipe wall, the pressure response decreased toward the unloaded level. These opposite response trends indicate that the pressure-sensing units can distinguish contact-enhancement defects caused by local narrowing from contact-reduction defects caused by wall-thinning corrosion.

The spatiotemporal response of the distributed sensing array was further analyzed. The rear pressure sensor array (P5–P8) exhibited similar response patterns to the front array (P1–P4), but with a clear time delay. This delay is consistent with the axial spacing between the two sensing arrays and the moving speed of the flexible tube. Therefore, the delayed responses of the front and rear arrays can provide positional information for tracking defect locations during continuous pipeline navigation.

The sensing behavior under more complex contact and deformation conditions was also evaluated. When local contact occurred at specific circumferential positions, such as near P2 or P4, the corresponding pressure channels showed clear step-like increases. At the same time, the bending channels exhibited differential responses related to the deformation state of the flexible tube. For example, B2 increased under tensile deformation on the outer side of the bend, whereas B4 decreased under compressive deformation on the inner side. B1 and B3 showed relatively smaller variations, as shown in Figure 9c. These results demonstrate that the pressure channels identify localized contact events, while the bending channels provide complementary information on tube posture and deformation during pipeline navigation.

To further examine the robustness of the sensing strategy under practical deployment conditions, supplementary validation experiments were performed and are provided in Figures S4 and S5. As shown in Figure S4, pipe diameter mismatch introduced preload-dependent baseline variations; however, defect-related differential responses remained distinguishable after baseline normalization. Figure S5 further evaluates the influence of axial rotation of the flexible tube. Controlled axial rotation mainly caused predictable migration of dominant responses among circumferential sensing channels while preserving defect detectability. Specifically, axial twisting changed the correspondence between the sensor-fixed channels and the pipe-fixed spatial directions, leading to sequential migration of both bending and pressure responses. These results indicate that rotation-induced signals correspond to deterministic channel redistribution rather than random false defect signals. Nevertheless, a complete real-time channel-remapping model for absolute pipe-fixed localization remains a future research direction.

## 4. Discussion

To further clarify the practical positioning and sensing precision of the proposed device relative to existing pipeline inspection methods, a comparative summary is provided in Table S1. The proposed flexible sensorized tube provides a multimodal sensing strategy for localized inspection in confined and curved pipeline environments. By integrating LIG-based pressure and bending sensing units on a flexible FPC–PDMS platform, the device can conform to narrow pipe geometries while continuously recording contact and deformation responses during navigation. Compared with rigid inspection probes or single-mode sensing devices, the circumferentially distributed pressure–bending architecture offers improved adaptability and richer mechanical information for identifying local morphological abnormalities.

The voltage signals obtained in this work should be interpreted as the contact-induced responses of the flexible tube rather than direct measurements of pipe-wall stress or strain. Local pipe morphology changes the mechanical interaction between the tube and the inner wall. A protrusion or dented region increases radial constraint and therefore produces an enhanced pressure response, whereas wall-thinning or corrosion-induced recession weakens local contact support and leads to a reduced pressure response. On this basis, the sensing output can be correlated with defect-induced geometric changes through controlled calibration. The tests with predefined dent depths and wall-thickness-reduction levels show distinguishable signal variations, indicating the feasibility of relative defect identification and semi-quantitative severity comparison.

The four-channel circumferential layout further improves the reliability of signal interpretation. Local defects generally produce channel-specific responses, while global disturbances such as preload variation, friction, or diameter mismatch mainly appear as correlated baseline changes. Therefore, multi-channel comparison and baseline normalization can help distinguish localized abnormal responses from common-mode interference. The bending channels also provide complementary information on tube deformation, which is useful for interpreting signals in curved or constrained pipeline sections.

Several limitations remain. The current system does not directly quantify pipe stress, strain, exact corrosion depth, or absolute wall-thickness loss. In addition, axial twisting during navigation may change the correspondence between the sensor-fixed channels and pipe-fixed spatial directions. Although the observed twisting-induced response migration is deterministic rather than random, real-time channel remapping has not yet been fully implemented. Future work will focus on quantitative defect calibration, rotational-state estimation, channel-remapping algorithms, and larger-scale in-pipe validation to improve absolute localization and practical deployment capability.

## 5. Conclusions

At present, pipeline inspection is mainly based on closed-circuit television systems or single-parameter physical sensing methods. These approaches are often insufficient for complex operational environments, especially in narrow urban pipelines with high curvature. In such conditions, a lack of haptic sensing makes it difficult to reliably identify interaction events, which increases the risk of misinterpretation or loss of critical tactile information.

To address these limitations, this paper proposes a low-cost flexible sensorized tube with a minimalist structural design. The device is capable of real-time multidirectional pressure sensing and bending posture acquisition, enabling more complete physical interaction perception within confined pipeline spaces. Compared with conventional approaches, it provides a more effective approach for supporting structural health monitoring in urban infrastructure.

The sensing device is designed for potential applications including remote inspection and pipeline condition assessment. By integrating pressure and bending sensing units on a flexible tubular platform, the device can provide complementary contact and deformation information in narrow and curved pipeline sections.

Experimental results demonstrate that the proposed system can effectively differentiate bending-related responses from localized pressure-induced signals through geometry-assisted signal differentiation. Stable sensing performance was maintained under representative pipeline defects, variable motion speeds, and curved pipeline conditions, demonstrating the feasibility of the proposed sensing strategy for flexible pipeline inspection applications.

## Data Availability

The original contributions presented in this study are included in the article/Supplementary Materials. Further inquiries can be directed to the corresponding author.

## Supplementary Materials

The following supporting information can be downloaded at https://www.mdpi.com/article/10.3390/s26113400/s1, Figure S1. Photographs of the fabricated sensing device and key components; Figure S2. Additional characterization of the pressure-sensing channel; Figure S3. Additional characterization of the bending sensing channel; Figure S4. Influence of pipe diameter mismatch on sensing performance; Figure S5. Axial-twisting-induced circumferential channel migration and pressure-channel transfer of the flexible sensorized tube; Table S1. Comparison of the proposed method with representative conventional pipeline inspection. References [42,43,44,45,46,47] are cited in the Supplementary Materials.

## References

[1] Jeon M., Choi M., Choi W., Ha J.M., Oh H. (2025). Near-Surface Defect Detection in Ultrasonic Testing Using Domain-Knowledge-Informed Self-Supervised Learning. Ultrasonics.

[2] Yu Y., Safari A., Niu X., Drinkwater B., Horoshenkov K.V. (2021). Acoustic and ultrasonic techniques for defect detection and condition monitoring in water and sewerage pipes: A review. Appl. Acoust..

[3] Zang X., Xu Z.-D., Lu H., Zhu C., Zhang Z. (2023). Ultrasonic guided wave techniques and applications in pipeline defect detection: A review. Int. J. Press. Vessel. Pip..

[4] Nguyen C.L., Nguyen A., Brown J., Dang L.M. (2025). Sewer pipeline condition assessment and defect detection using computer vision. Autom. Constr..

[5] Nie H., Hao F., Wang L., Guo Q., Chen H., Ren J., Wang K., Dang W., Liang X., Ma W. (2023). Application of Terahertz Nondestructive Testing Technology in the Detection of Polyethylene Pipe Defects. ACS Omega.

[6] Waqar M., Louati M., Ghidaoui M.S. (2023). Time-Reversal Technique for Pipeline Defect Detection. Water Res..

[7] Christensen S.L., Pereyra M., Riis N.A.B., Jørgensen J.S. (2024). A Bayesian approach for CT reconstruction with defect detection for subsea pipelines. Inverse Probl..

[8] Rayhana R., Yun H., Liu Z., Kong X. (2024). Automated defect-detection system for water pipelines based on CCTV inspection videos of autonomous robotic platforms. IEEE/ASME Trans. Mechatron..

[9] Chen H., Song K., Tan Y., Xue X., Yan Y., Tao X., Shen F. (2025). Float in the air: Defect detection in pipelines with electromagnetic levitation technology. IEEE/ASME Trans. Mechatron..

[10] Yussuf A.N., Weerasinghe N.P., Chen H., Hou L., Herath D., Rashid M., Zhang G., Setunge S. (2025). Leveraging Deep Learning Techniques for Condition Assessment of Stormwater Pipe Network. J. Civ. Struct. Health Monit..

[11] Su T.-C., Yang M.-D. (2014). Application of morphological segmentation to leaking defect detection in sewer pipelines. Sensors.

[12] Wu B., Zhou W. (2023). Ultrasonic Defect Detection in Noisy Signals by a Nonconvex Sparse Regularization Approach. Appl. Acoust..

[13] Feng J., Li Q., Xiao Q., Wang G. (2023). A Method of Rayleigh Wave Combined With Coil Spatial Pulse Compression Technique for Crack Defects Detection. IEEE Trans. Instrum. Meas..

[14] Hawari A., Alamin M., Alkadour F., Elmasry M., Zayed T. (2018). Automated Defect Detection Tool for Closed Circuit Television (Cctv) Inspected Sewer Pipelines. Autom. Constr..

[15] Jung M., Park B., Bae J., Shin S. (2018). PAUT-Based Defect Detection Method for Submarine Pressure Hulls. Int. J. Nav. Archit. Ocean. Eng..

[16] Casavola C., Palano F., De Cillis F., Tati A., Terzi R., Luprano V. (2018). Analysis of CFRP Joints by Means of T-Pull Mechanical Test and Ultrasonic Defects Detection. Materials.

[17] Wang Y., Han S., Yu Y., Qi X., Zhang Y., Lian Y., Bai Z., Wang Y., Lv Z. (2021). Numerical Simulation of Metal Defect Detection Based on Laser Ultrasound. IEEE Photonics J..

[18] Zhang L., Cameron I.M., Ledger P.D., Belblidia F., Pearson N.R., Charlton P., Sienz J. (2023). Effect of Scanning Acceleration on the Leakage Signal in Magnetic Flux Leakage Type of Non-Destructive Testing. J. Nondestruct. Eval..

[19] Martinenq J.-P. Inertial Measurement Unit for Trajectography. Proceedings of the 2021 2nd International Conference on Range Technology (ICORT).

[20] Mogatadakala K.V., Zhang Z. (2013). Phase Errors in FSE Signals Due to Low Frequency Electromagnetic Interference. Magn. Reson. Imaging.

[21] Yang R., Singh S.K., Tavakkoli M., Karami M.A., Rai R. (2023). Deep Learning Architecture for Computer Vision-Based Structural Defect Detection. Appl. Intell..

[22] Arciniegas A., Achdjian H., Bustillo J., Vander Meulen F., Fortineau J. (2017). Experimental Simultaneous Measurement of Ultrasonic Properties and Thickness for Defect Detection in Curved Polymer Samples. J. Nondestruct. Eval..

[23] Wang H.L., Kuang S.Y., Li H.Y., Wang Z.L., Zhu G. (2020). Large-Area Integrated Triboelectric Sensor Array for Wireless Static and Dynamic Pressure Detection and Mapping. Small.

[24] Liu Y., Wang H., Zhao W., Qin H., Fang X. (2016). Thermal-Performance Instability in Piezoresistive Sensors: Inducement and Improvement. Sensors.

[25] Zeng Y., Zou Y., Lu X., Zhou C., Zhao M., Du T., Yuan H., Xu M. (2026). Advances in Triboelectric Sensor in Extremely Harsh Environments. Small Methods.

[26] Zhu M., He T., Lee C. (2020). Technologies toward next Generation Human Machine Interfaces: From Machine Learning Enhanced Tactile Sensing to Neuromorphic Sensory Systems. Appl. Phys. Rev..

[27] Li M., Wang T., Han C., Yang H., Huang Y., Hu J., Li L., Jiang J., Huang M., Fan Y. (2024). A Novel Piezoresistive Sensor with Rectification Properties. Mater. Des..

[28] Niu D., Jiang W., Ye G., Wang K., Yin L., Shi Y., Chen B., Luo F., Liu H. (2018). Graphene-Elastomer Nanocomposites Based Flexible Piezoresistive Sensors for Strain and Pressure Detection. Mater. Res. Bull..

[29] Mahdi Z.K., Abbas R.A., Al-Taleb M.K.H., Ali A.H., Mohamed E.M. (2024). Upgrading Sustainable Pipeline Monitoring with Piezoelectric Energy Harvesting. Processes.

[30] Wang L., Wang M., Lu J., Ardhi R.E.A., Liu J., Liu G., Lee J.K. (2019). Enhanced Adhesion between Liquid Metal Ink and the Wetted Printer Paper for Direct Writing Electronic Circuits. J. Taiwan Inst. Chem. Eng..

[31] Pavel I.-A., Lakard S., Lakard B. (2022). Flexible Sensors Based on Conductive Polymers. Chemosensors.

[32] Ning C., Gong D., Wu L., Chen W., Zhang C. (2024). Preparation of Gradient Polyurethane and Its Performance for Flexible Sensors. Polymers.

[33] Levi A., Piovanelli M., Furlan S., Mazzolai B., Beccai L. (2013). Soft, Transparent, Electronic Skin for Distributed and Multiple Pressure Sensing. Sensors.

[34] Zhou Z., Jin Y., Fu J., Si S., Liu M., Hu Y., Gan J., Deng Y., Li R., Yang J. (2025). Smart Wireless Flexible Sensing System for Unconstrained Monitoring of Ballistocardiogram and Respiration. npj Flex. Electron..

[35] Gupta P., Panda T., Allu S., Borah S., Baishya A., Gunnam A., Nangia A., Naumov P., Nath N.K. (2019). Crystalline Acylhydrazone Photoswitches with Multiple Mechanical Responses. Cryst. Growth Des..

[36] Jiang H., Chen D., Bai H., Li G., Luo X., Ji Z., Wen G., Huang Y. Research and Implementation of Parallel Data Acquisition System for Inertial Sensor Array. Proceedings of the 2024 IEEE 7th International Conference on Electronic Information and Communication Technology (ICEICT).

[37] Xu Y., Wu Z., Li B., Deng S., Zhong W., Li G., Luo D., Sze Yan Yeung F., Kwok H.S., Chen R. (2021). Oxide TFT Frontend Amplifiers for Flexible Sensing Systems. IEEE Trans. Electron Devices.

[38] Liu F., Luo S., Li J., Wang Z., Wang X., Hao W., Wang Y. (2025). Laser-Induced 3D Graphene Enabled Polymer Composites with Improved Mechanical and Electrical Properties Toward Multifunctional Performance. Adv. Sci..

[39] Machado B.S., Morais M., Pinheiro T., Deuermeier J., Teixeira V., Nunes D., Martins R., Inácio J.M., Fortunato E., Almeida H.V. (2025). One-Step Production of Laser-Induced Graphene via CO_2_ Laser on Agarose–Lignin Membranes. Flex. Print. Electron..

[40] Huang L., Su J., Song Y., Ye R. (2020). Laser-Induced Graphene: En Route to Smart Sensing. Nano-Micro Lett..

[41] Wanjari V.P., Reddy A.S., Duttagupta S.P., Singh S.P. (2022). Laser-Induced Graphene-Based Electrochemical Biosensors for Environmental Applications: A Perspective. Environ. Sci. Pollut. Res..

[42] Cao J., Zhong J., Li J., Luo Z., Zhang H., Wang Q. (2017). Laser Ultrasonic Pipeline Detection System Based on LabVIEW. China Meas. Test..

[43] Liu R., Shao Z., Sun Q., Yu Z. (2024). Defect Detection and 3D Reconstruction of Complex Urban Underground Pipeline Scenes for Sewer Robots. Sensors.

[44] Fan X., Chai N., Shi Y., Chen Y., Chen F., Ye W. (2025). Application and Progress of Electromagnetic Testing Technology in Weld Defect Detection for Oil and Gas Pipelines. Nondestruct. Test..

[45] Yang P. (2021). Application of Ultrasonic Guided Wave Testing Technology in Pressure Pipeline Inspection. Chem. Enterp. Manag..

[46] Zeng T., Chai S., Xu J., Xu S. (2026). A Curvature-Adaptive Flexible Capacitive Sensing System for Early Detection of Corrosion under Insulation in Long-Distance Thermal Pipelines. Sci. Rep..

[47] Wang J., Wan L., Chen K., Zhang T., Liu Z., Fu Z., Ni J., Gao Z., Fu H. (2026). Experimental Investigation on Buried Pipeline Bending Deformation Monitoring Using Flexible Piezoelectric Sensing. Tunn. Undergr. Space Technol..

