# Supplementary material for: Flexible Sensorized Tube for Pipeline Defect Detection Based on Bending and Pressure Sensing
Source: Sensors (Basel). 2026 May 27;26(11):3400. doi: 10.3390/s26113400 (PMC13259390; doi:10.3390/s26113400)
Supplement: Supplementary file 1 [file sensors-26-03400-s001.zip › sensors-4294694-supplementary.pdf]

Supplementary File

Flexible Sensorized Tube for Pipeline Defect Detection Based on Bending and Pressure Sensing

Yikang Chen [1], Hongyuan Chen [2], Yuan Yin [1], Junyi Chen [2], Bo Lu [2], Tao Chen [1] and Minglu Zhu [2,*]

[1] School of Future Science and Engineering, Soochow University, Suzhou 215123, China; 20234229006@stu.suda.edu.cn (Y.C.); 20234229037@stu.suda.edu.cn (Y.Y.)

[2] Jiangsu Provincial Key Laboratory of Advanced Robotics, School of Mechanical and Electric Engineering,China; 20255229162@stu.suda.edu.cn (H.C.); 20245229045@stu.suda.edu.cn(J.C.); blu@suda.edu.cn (B.L.); chent@suda.edu.cn (T.C.);

[*] Correspondence: mlzhu@suda.edu.cn (M.Z.)

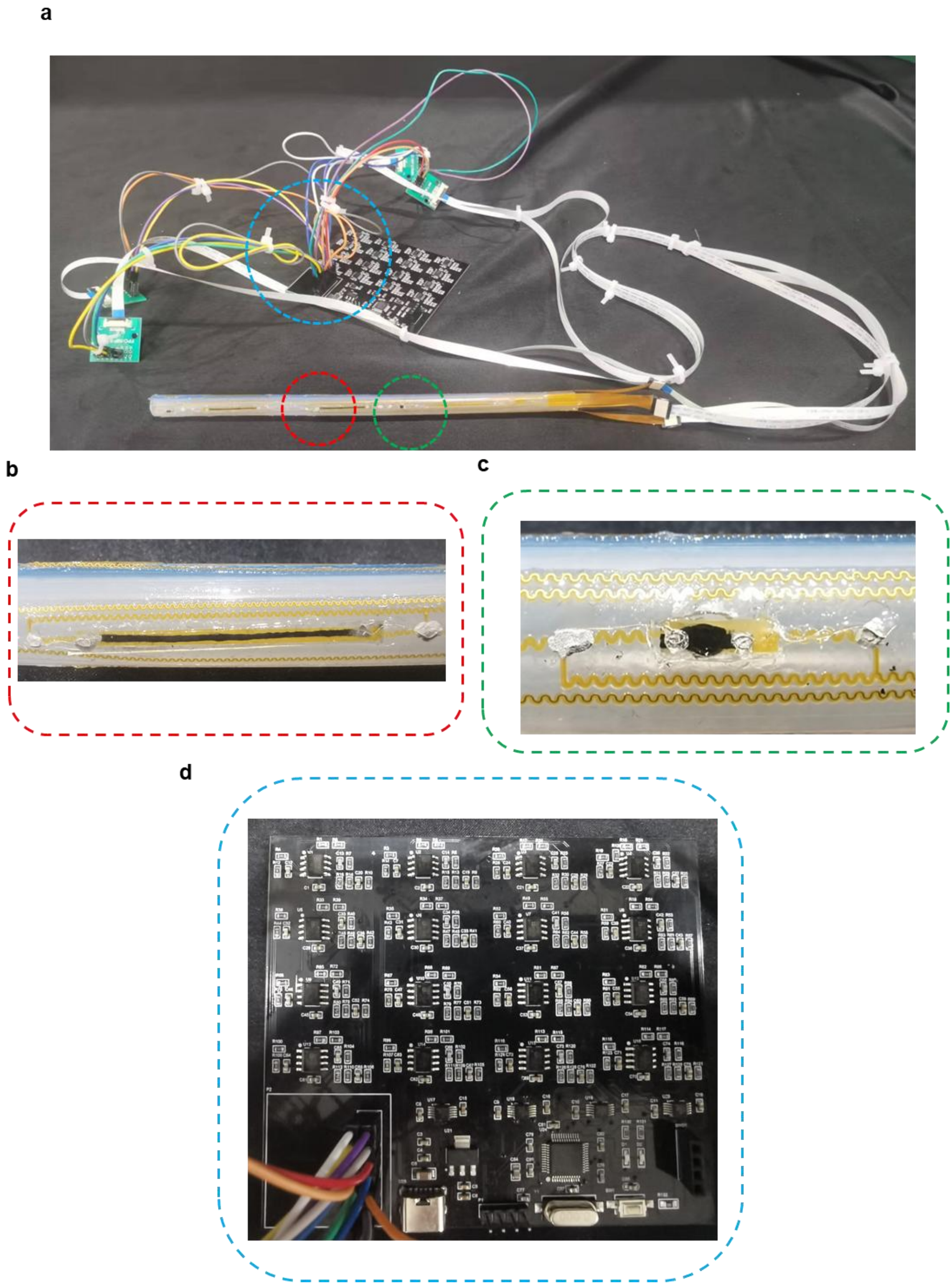

Figure S1. Photographs of the fabricated sensing device and key components. (a) Flexible sensorized tube integrating circumferential pressure and bending sensing modules. (b) Photograph of the fabricated bending sensor. (c) Photograph of the fabricated pressure sensor. (d) Photograph of the custom PCB signal acquisition board used for sensor readout and data collection.

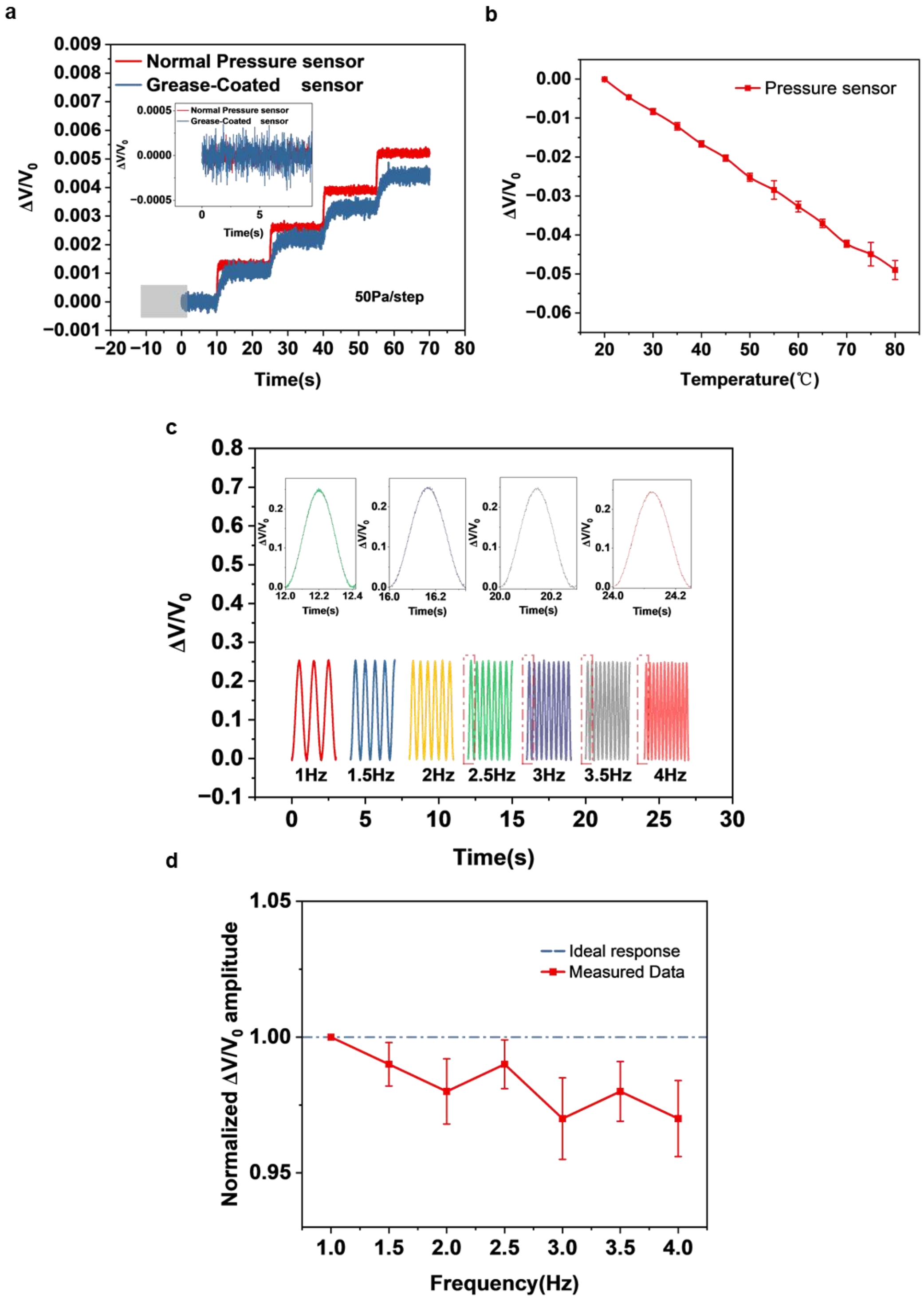

Figure S2. Additional characterization of the pressure sensing channel. (a) Resolution and baseline noise characterization of the pressure sensor under incremental low-pressure loading. Clear and stable signal plateaus are observed, demonstrating practical pressure resolution and low baseline fluctuation. (b) Temperature drift characterization of the pressure sensor under ambient temperature variation from 25 °C to 75 °C. (c) Representative pressure sensing waveforms under cyclic loading at different frequencies. (d) Frequency-dependent normalized response amplitude of the pressure sensor, showing stable dynamic performance across the tested frequency range.

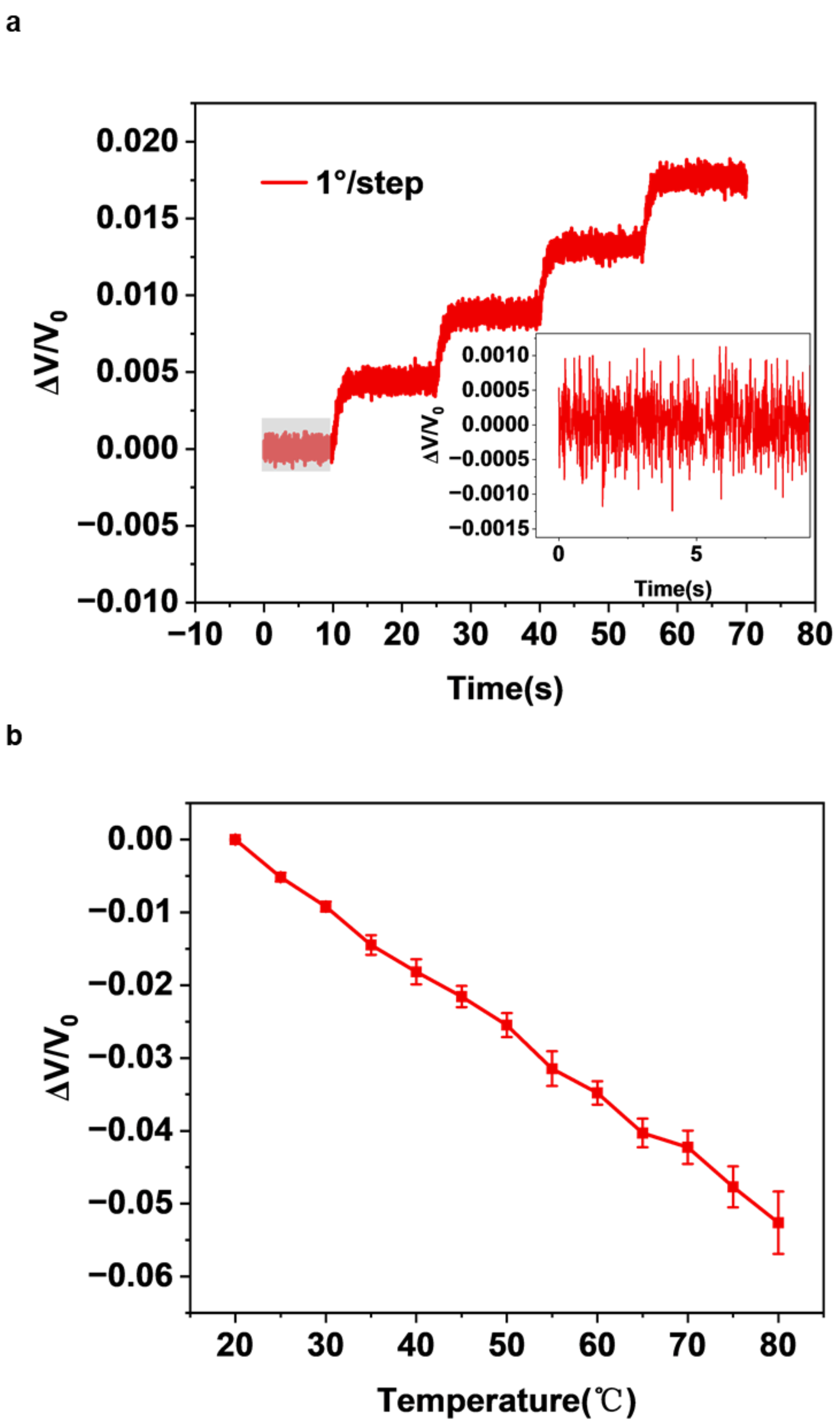

Figure S3. Additional characterization of the bending sensing channel. (a) Strain resolution and baseline noise characterization of the bending sensor under incremental tensile strain loading, demonstrating stable signal discrimination of small deformation changes. (b) Temperature drift characterization of the bending sensor under ambient temperature variation from 25 °C to 75 °C.

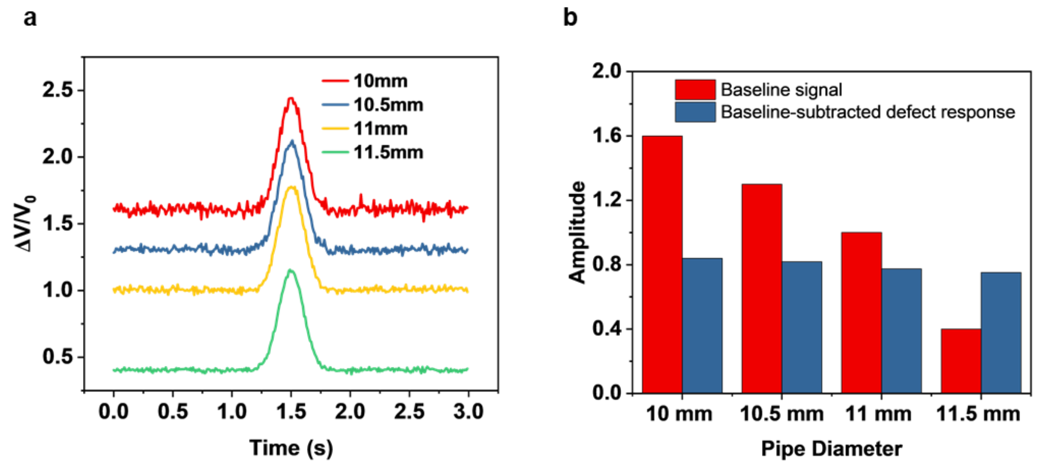

Figure S4. Influence of pipe diameter mismatch on sensing performance. (a) Pressure responses measured under different pipe inner diameters corresponding to 10 mm, 10.5 mm, 11 mm, and 11.5mm fitting conditions. Smaller pipe diameters increase radial preload and friction, resulting in elevated baseline signals. (b) Comparison of baseline signals and baseline-subtracted defect responses under different diameter conditions.

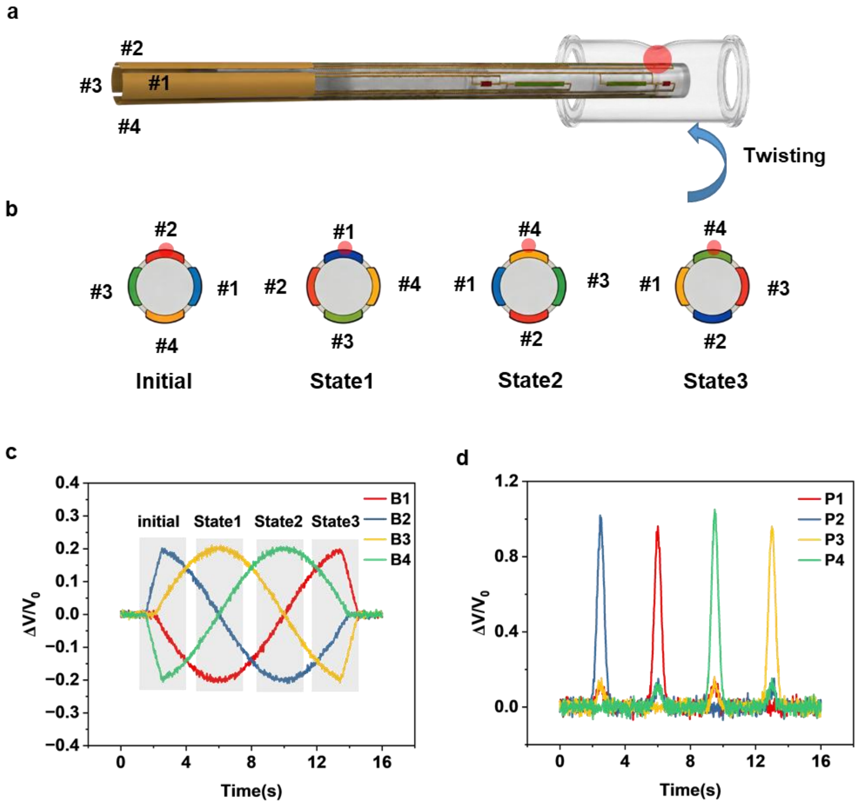

Figure S5. Axial-twisting-induced circumferential channel migration and pressure-channel transfer of the flexible sensorized tube. (a) Schematic illustration of the flexible sensorized tube undergoing axial twisting during pipe inspection. The local contact position remains fixed in the pipe-fixed coordinate system, while the circumferential sensing channels rotate with the tube. (b) Cross-sectional schematics of the four circumferential sensing channels under different twisting states. (c) Representative bending responses of B1–B4 during axial twisting. (d) Representative pressure responses of P1–P4 under localized contact.

Table S1. Comparison of the proposed method with representative conventional pipeline inspection methods.

Method	Target Parameter	Pressure / Load Resolution	Spatial / Deformation Resolution	Measurement Range	Signal Differentiation Capability
Laser Profiling / Optical Point-cloud Reconstruction [1] [2]	Surface geometry and internal profile mapping	N/A	Sub-millimeter profile reconstruction (~0.1 mm)	Limited by line-of-sight and pipe diameter constraints	None. Only provides static geometric reconstruction without tactile information.
Ultrasonic / Guided Wave Ultrasonic Testing (UT/GWUT) [3] [4]	Wall thickness loss and structural cracks	N/A	~0.1 mm wall-thickness resolution	Restricted by signal attenuation and complex wave scattering in highly curved pipelines	Limited. Requires coupling medium, and signal interpretation becomes challenging in complex bends.
Flexible Coplanar Capacitive Sensing (Zeng et al.) [5]	Dielectric voids / insulation defects	N/A	Detects subsurface defects up to 50 mm depth	Depends on optimized lift-off height (~1 mm)	Limited. Signal aliasing between surface morphology and internal defects may occur.
Piezoelectric PVDF Geocable (Wang et al.) [6]	Dynamic strain and deformation rate	Indirect. Voltage output correlated with loading rate	Strain accuracy of ±10 με	Up to 7.71% strain	Moderate. Sensitive to deformation but affected by directional asymmetry and compression instability.
This Work (Geometry-differentiated flexible array)	Simultaneous pressure and bending sensing	~50 Pa (direct pressure sensing resolution)	Reliable discrimination under 10° incremental bending	0–40% strain / accommodates severe 70°	Good. Geometry-assisted signal differentiation reduces pressure–bending interference and improves multimodal signal interpretability under confined pipeline conditions.

References

1. Cao, J.; Zhong, J.; Li, J.; Luo, Z.; Zhang, H.; Wang, Q. Laser Ultrasonic Pipeline Detection System Based on LabVIEW. China Meas. Test. 2017, 43, 80–85.
2. Liu, R.; Shao, Z.; Sun, Q.; Yu, Z. Defect Detection and 3D Reconstruction of Complex Urban Underground Pipeline Scenes for Sewer Robots. Sensors 2024, 24, 7557.
3. Fan, X.; Chai, N.; Shi, Y.; Chen, Y.; Chen, F.; Ye, W. Application and Progress of Electromagnetic Testing Technology in Weld Defect Detection for Oil and Gas Pipelines. Nondestruct. Test. 2025, 47, 79–85.
4. Yang, P. Application of Ultrasonic Guided Wave Testing Technology in Pressure Pipeline Inspection. Chem. Enterp. Manag. 2021, 30, 67–68.
5. Zeng, T.; Chai, S.; Xu, J.; Xu, S. A Curvature-Adaptive Flexible Capacitive Sensing System for Early Detection of Corrosion under Insulation in Long-Distance Thermal Pipelines. Sci Rep 2026.
6. Wang, J.; Wan, L.; Chen, K.; Zhang, T.; Liu, Z.; Fu, Z.; Ni, J.; Gao, Z.; Fu, H. Experimental Investigation on Buried Pipeline Bending Deformation Monitoring Using Flexible Piezoelectric Sensing. Tunnelling and Underground Space Technology 2026, 168, 107218.
